# Dr. Granger’s Book on Care of the Insane

**Published:** 1886-06

**Authors:** 


### Dr. Granger's Book on Care of the Insane.

We have just received a copy of Dr. Granger's book on this subject. Dr. Granger was among the first to conceive the idea that to properly interest attendants and employees in asylums in their most arduous work, was to teach them that the insane are sick and irresponsible persons, and they should thus be treated. This idea, with the help of the other physicians in the Buffalo State Asylum, he has thoroughly imbued into the minds of the attendants, and great has been the reward. To the doctor and his associates are due the highest praise for the labor performed and the good accomplished. He now seeks to present to every asylum the same advantages which this one enjoys, by offering to them this little manual, which contains full instructions for carrying on the work. This book should find its way into the hands of every asylum attendant who desires to elevate himself and his calling.

